# Factors associated with anti-severe acute respiratory syndrome coronavirus 2 (SARS-CoV-2) spike protein antibody titer and neutralizing activity among healthcare workers following vaccination with the BNT162b2 vaccine

**DOI:** 10.1371/journal.pone.0269917

**Published:** 2022-06-10

**Authors:** Yurie Kobashi, Yuzo Shimazu, Takeshi Kawamura, Yoshitaka Nishikawa, Fumiya Omata, Yudai Kaneko, Tatsuhiko Kodama, Masaharu Tsubokura

**Affiliations:** 1 Department of General Internal Medicine, Hirata Central Hospital, Hirata, Ishikawa District, Fukushima, Japan; 2 Department of Radiation Health Management, Fukushima Medical University School of Medicine, Fukushima City, Fukushima, Japan; 3 Southern Tohoku Research Institute for Neuroscience, Yatsuyamada, Koriyama, Fukushima, Japan; 4 Proteomics Laboratory, Isotope Science Center, The University of Tokyo, Tokyo, Japan; 5 Laboratory for Systems Biology and Medicine, Research Center for Advanced Science and Technology, The University of Tokyo, Tokyo, Japan; Universitas Indonesia Fakultas Kedokteran, INDONESIA

## Abstract

The purpose of this study was to identify factors associated with the increase in the severe acute respiratory syndrome coronavirus 2 (SARS-CoV-2) spike (S1) protein and neutralizing antibody titer following SARS-CoV-2 vaccination. This observational study was conducted among healthcare workers working for a private hospital group in Fukushima Prefecture, Japan. Two blood samples were obtained from each participant. The first sample was obtained before the first dose of BNT162b2 (Pfizer-BioNTech) vaccine, and a second sample was obtained approximately 6 weeks later. Immunoglobulin G (IgG) antibody against the SARS-CoV-2 spike (S1) protein, immunoglobulin M (IgM) antibody against SARS-CoV-2 N-protein, and neutralizing activity were measured using the chemiluminescent immunoassay with iFlash 3000. A total of 231 healthcare workers who agreed to participate, and were negative for anti-SARS-CoV-2 IgM antibodies at enrollment, were included in the analysis. All participants had elevated IgG antibodies and neutralizing activity above the cutoff values. A total of 174 (75.3%) and 208 (90.0%) participants experienced adverse reactions after the first and second vaccine doses, respectively. Younger age, female sex, not taking immunosuppressive or antipyretic analgesic medication regularly, a lack of local adverse reactions after the first dose, and the presence of adverse reactions (fever, muscle, and joint pain) after the second dose were associated with higher IgG antibody titers and neutralizing activity. Intake of analgesic antipyretic for adverse reactions to vaccines was not significantly associated with antibody and neutralizing activity titer production. Immune responses after vaccination may differ among individuals, and continued countermeasures to prevent SARS-CoV-2 infection are vital.

## Introduction

Coronavirus disease (COVID-19) vaccination is being performed worldwide to control the COVID-19 pandemic. Various issues, such as shortage of vaccines [1], amplification of Delta strains that reduce the efficacy of vaccines [2], and individual differences in the acquisition of immunity after vaccines [3, 4], have not been addressed. When considering strategy-related vaccination, assays such as antibody titer and neutralizing activity to clarify the status of immunity after vaccination may help solve these challenges. Therefore, accumulating evidence on immunity after vaccination, such as antibody titers, is vital.

To date, studies on antibody titers after vaccination have shown that two doses of vaccine are effective against severe acute respiratory syndrome (SARS-CoV-2) infection [5, 6]. However, other studies have shown that the acquisition of immunity and antibodies after vaccination may differ according to the brand of vaccine [7] and the antibody titer is affected by the interval between vaccinations [8]. In particular, a previous study showed that antibody titers were lower among elderly and men [3], and that daily intake of immunosuppressant medicine and alcohol were associated with lower antibody titers [5]. In addition, solid organ transplantation and hematopoietic stem-cell transplant recipients have lower antibody titers than healthy individuals [4, 9, 10]. However, the number of studies on factors affecting antibody titer after vaccination is limited, especially studies using multiple outcome measures, such as antibody titer and neutralizing activity.

In Japan, healthcare workers (HCWs) are prioritized to receive vaccination. As of July 31, 2021, there were 925,823 COVID-19 cases in Japan, and the number of patients is still increasing [11]. In addition, the nationwide shortage of vaccines is a crucial problem. A similar situation occurs in rural Fukushima Prefecture, where medical resources are remarkably limited. As of July 31, 2021, there were 5833 individuals infected with COVID-19 in Fukushima prefecture, and the number of patients is still increasing [12]. Thus, it is vital to determine immunity after vaccination among HCWs in this area. Moreover, antibody titers of HCWs have been continuously examined since last year in the Ken-chu District of Fukushima Prefecture, and this information has been accumulating [13–15]. Hence, this cohort in the Ken-chu District of Fukushima Prefecture is a good population to examine factors affecting antibody titers and neutralizing activity.

The purpose of this study was to identify factors that influence the increase in SARS-CoV-2 antibody titers, including antibodies against the spike (S1) protein and neutralizing activity, following SARS-CoV-2 vaccination in the Ken-chu District of Fukushima Prefecture. Medical resources in the prefecture are limited; hence, prevention of infection is the main measure for infection control. The individual factors considered were age, sex, medication use, medical history, and adverse reactions after the first and second doses of vaccine.

## Methods

This was an observational study of HCWs working for Seireikai, a private hospital group that includes the Hirata Central Hospital, clinics, nursing homes, and daycare centers in the Ken-chu District, Fukushima Prefecture, Japan. The first dose of vaccination was administered in April 2021, and the second dose was administered in May, 21 days after the first dose. The study observed routine hospital vaccinations.

### Procedure

To identify the factors that affect the antibody titers and neutralizing activity, blood samples were obtained twice among the hospital staff. The first blood sample was obtained between April 19 and 23, 2021, before the BNT162b2 (Pfizer-BioNTech) vaccine was administered. The second blood sample was obtained between May 31 and June 6, 2021, 21 days after the second dose of vaccine was administered (6 weeks after the first blood draw). The blood samples were obtained by medical staff of the Seireikai Group. Approximately 9 mL of blood was obtained each time. The samples were sent to the University of Tokyo for testing by the staff of the Seireikai Group. Participant background information was retrieved from their medical records, and additional information, including medical history, daily medication, and adverse reactions after vaccination, were obtained from a questionnaire. The study was approved by the ethics committees of Hirata Central Hospital (number 2021-0611-1) and Fukushima Medical University (number 2021–116).

### Laboratory analysis

Immunoglobulin G (IgG) antibody against the SARS-CoV-2 spike (S1) protein, immunoglobulin M (IgM) antibody against SARS-CoV-2 nucleocapsid (N) protein, and neutralizing activity were measured using the CLIA assay with iFlash 3000 (YHLO Biotech, Shenzhen, China) at the University of Tokyo between June 9 and 16, 2021. IgM antibody against SARS-CoV-2 N-protein was measured in the first blood sample to determine the infection status and past infection history. IgG antibodies were measured in the first and second blood samples to determine the change in antibody titers between the first vaccine dose and 21 days after the second dose vaccination. Neutralizing activity was measured in the second blood sample to assess the effectiveness of vaccinations. The cutoff values of IgG, IgM, and neutralizing activity were 10 arbitrary units per milliliter (AU/mL), and the tests were performed according to the manufacturer’s guidelines. A quality check was conducted daily before the samples were tested. The expected value and the confidence range of the calibration reagent for each lot were provided by the manufacturer, and the participant samples were tested after confirming that the calibration reagent values were within the reference range [13].

### Statistics analysis

A descriptive analysis was conducted. Categorical variables were summarized as frequencies and percentages, and antibody titers were summarized as medians and interquartile ranges. A multivariable linear regression analysis was performed to determine the factors associated with elevation of IgG antibody titers and neutralizing activity. The model included age, sex, daily medication (i.e., immunosuppressive drugs, antiallergic drugs, and antipyretic analgesics), adverse reactions, and measures of adverse reactions (local adverse reaction, fever >37.5°C, muscle and joint pain, fatigue, and headache) as independent variables, which were chosen based on a literature review. A statistically significant p-value was set at 0.05. STATA IC (version 15, Lightstone, TX, USA) was used for all analyses.

## Results

A total of 235 hospital staff provided blood samples, of whom 231 (98.3%) provided two blood samples, agreed to participate in the study, and were negative for IgM against N protein antibodies, were included in the analysis. The vaccination interval was 21 days among all participants. The median interval between the first and second blood sample collections was 42 days (Table 1).

**Table 1:** Characteristics of the participants (n = 231).

	n	%
**Sex**		
Male	63	27.3
Female	168	72.7
**Age (years)**		
18–44	119	51.5
45–64	96	41.6
65–78	16	6.93
**Occupation**		
Doctor	3	1.30
Nurse	60	26.0
Caregiver	60	26.0
Other medical staff	39	16.9
Office worker	28	12.1
Other non-medical staff	41	17.8
**Underlying disease**		
Yes	75	32.5
None	156	67.5
**IgG S1 (before vaccination)**, median [IQR]	0.33 [0.28–0.41]	
**IgG S1 (after vaccination)**, median [IQR]	1740 [1201–2185]	
**Neutralizing activity**, median [IQR]	703 [583–779]	
**Daily medicine**		
Immunosuppressive drug	2	0.86
Antiallergic drug	7	3.02
Analgesic and antipyretic drug	14	6.03
**Measure for harmful aftereffects**		
Taking off from work	50	21.6
Taking medicine	52	22.4
Other	6	2.59
Nothing	139	59.9

IgG, immunoglobulin G; IgM, immunoglobulin M; IQR, interquartile range; S1, spike protein

**Fig 1:**
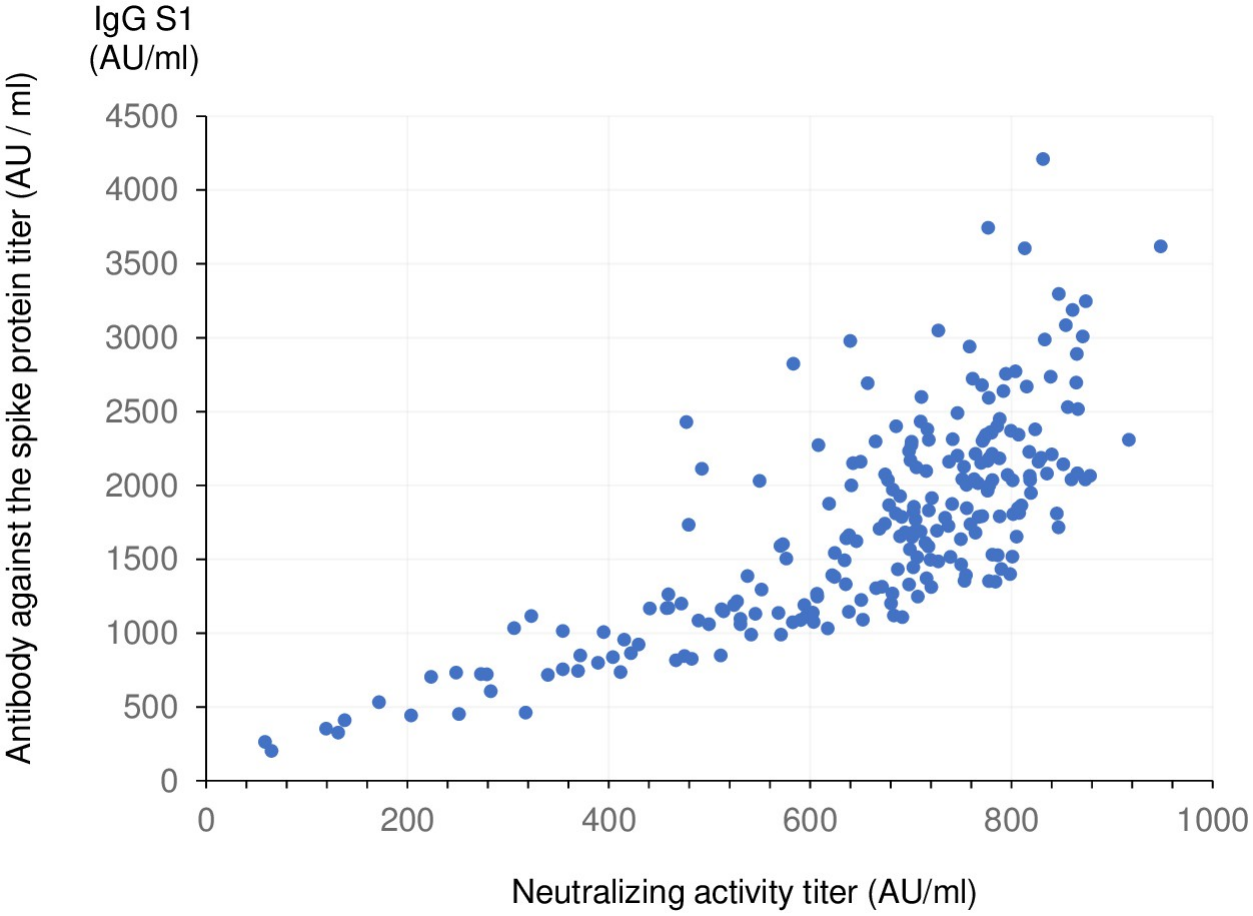
Anti-SARS-CoV-2 spike protein antibody titer and neutralizing activity.

IgG antibody titers and neutralizing activity were correlated (Fig 1). All participants had elevated IgG antibodies and neutralizing activity above the threshold values (S1 Fig).

Of the participants, 174 (75.3%) and 208 (90.0%) had adverse reactions after the first and second vaccine doses, respectively. A total of 147 (63.6%) and 144 (62.3%) of participants experienced local adverse reactions after the first and second vaccine doses, respectively. The major systemic adverse reactions (in order of frequency) were, fatigue, muscle and joint pain, and headache after the first vaccination; fatigue, headache, and muscle and joint pain after the second vaccination (Table 2).

**Table 2:** Adverse reactions.

	First dose		Second dose	
	n	%	n	%
Local pain, swelling, and redness	147	63.6	144	62.3
Fatigue	54	23.4	137	59.3
Fever <37.5°C	16	6.9	36	15.6
Fever ≥37.5°C	7	3.0	58	25.1
Muscle pain, joint pain	42	18.2	83	35.9
Headache	27	11.7	86	37.2
Nausea	8	3.5	12	5.2
Dizziness	7	3.0	11	4.8
Other	9	3.9	19	8.2
None	57	24.7	23	10.0

Factors associated with IgG against S1 protein antibody titer included age, regular intake of immunosuppressive and analgesic antipyretic medications, local adverse reactions after the first dose, and systemic adverse reactions (fever >37.5°C, muscle and joint pain) after the second dose. Factors associated with neutralizing activity included age, sex, regular intake of immunosuppressive and analgesic antipyretic medications, and systemic adverse reactions (muscle and joint pain) after the second dose. Intake of analgesic antipyretic for adverse reactions to vaccines was not significantly associated with antibody titer or neutralizing activity (Table 3).

**Table 3:** Multivariable linear regression analysis for IgG antibody against the SARS-CoV-2 spike protein titers and neutralizing activity.

	IgG antibody				Neutralizing activity			
	β	SE	Adjusted β	p value	β	SE	Adjusted β	p value
**Age**	−12.6	3.3	−0.241	**<0.001**	−3.2	0.8	−0.244	**<0.001**
**Female sex**	42.8	95.0	0.027	0.65	55.8	24.5	0.142	**0.024**
**First dose**								
Local pain, swelling, and redness	−253.8	110.3	−0.175	**0.022**	−23.5	28.4	−0.065	0.41
Fever over 37.5°C	−170.5	259.4	−0.042	0.51	37.8	66.8	0.037	0.57
Muscle pain, joint pain	42.3	127.9	0.023	0.74	−3.1	32.9	−0.007	0.93
Fatigue	29.4	117.9	0.018	0.80	−7.2	30.3	−0.017	0.81
Headache	−77.7	146.9	−0.036	0.60	−43.8	37.8	−0.080	0.25
**Second dose**								
Local pain, swelling, and redness	57.7	110.2	0.040	0.60	4.2	28.4	0.012	0.88
Fever over 37.5°C	473.7	112.3	0.294	**<0.001**	49.4	28.9	0.122	0.089
Muscle pain, joint pain	223.8	108.9	0.154	**0.041**	69.3	28.0	0.190	**0.014**
Fatigue	−7.6	101.5	−0.005	0.94	15.1	26.1	0.043	0.56
Headache	44.3	102.1	0.031	0.67	5.7	26.3	0.016	0.83
**Daily medication**								
Immunosuppressive drug	−1352.4	451.6	−0.179	**0.003**	−440.8	116.2	−0.233	**<0.001**
Antiallergic drug	−38.4	248.4	−0.009	0.88	−17.1	64.0	−0.017	0.79
Analgesic and antipyretic drug	−400.9	184.8	−0.137	**0.031**	−134.2	47.6	−0.183	**0.005**
**Drug for harmful aftereffects**	−64.7	112.6	−0.039	0.57	−22.6	29.0	−0.054	0.44

β, coefficient; SE, standard error

## Discussion

Laboratory testing of antibody titer and neutralizing activity toward SARS-CoV-2 following vaccination provide vital evidence regarding individual differences in immune response, and is useful for determining vaccination strategy. However, the number of studies on the association between immunity after vaccination and individual factors is limited, especially studies with multiple outcomes.

Younger age, not taking immunosuppressive medication or analgesic antipyretic medication regularly, and the presence of adverse reaction of muscle and joint pain after the second dose were associated with elevated both IgG against S1 protein antibody titer and neutralizing activity. A lack of local adverse reactions after the first dose and adverse reaction of fever after second dose were associated with high IgG against S1 protein antibody titer; in contrast, sex was associated with high neutralizing activity, For IgG antibodies, the presence of an adverse reaction of fever over 37.5°C after the second dose was the strongest factor associated with antibody titer elevation. For neutralizing activity, age was the most significant factor. Previous studies have found an association between daily intake of immunosuppressant and antiallergic medications and antibody levels [5]. While the association between antibody titers and protection from infection requires further evaluation [16], specific precautions may be required for individuals with low antibody titers.

Daily administration of antipyretic analgesics was significantly associated with a lower antibody titer, while taking a single-dose of antipyretic analgesic for adverse reactions after the vaccine had no association with antibody titers. These results are consistent with what is known regarding the association between IgG antibodies and neutralizing activity. In this study, 90% of the participants experienced adverse reactions after the second vaccination; thus, antipyretic analgesic medication might be useful for the relief of symptoms. Further research is required to determine the effect of different antipyretic analgesics on antibody titers; however, the blood study results did not find that the administration of antipyretic analgesic medicines as a therapy for adverse reactions after vaccination had an effect on the antibody response.

The IgG antibody titer and neutralizing activity substantially increased in all participants at 3 weeks after the second vaccination, although the antibody titer varied among individuals. The lowest neutralizing activity titer at 3 weeks after the second dose was 58 AU/mL, which was above the cutoff value (10 AU/mL). Previous studies have shown that the antibody titer is correlated with protection against infection [17]. To prevent clusters of cases of COVID-19 in hospitals and nursing homes, HCWs are required to prevent exacerbation of the disease and transmission of infection. Thus, HCWs with low antibody titers should be followed-up and further studies are required to determine the value of providing a third booster dose of vaccine.

This study had several limitations. First, the sample size was small. In addition, the small sample size of males might affect the multivariable linear regression analysis result. Second, it was reported by the manufacturer that the titer of neutralizing activity above 450 AU/mL might not be accurate because of the problem of correlation between luminescence and titer. Third, information on adverse reactions was obtained using a self-reported questionnaire survey; thus, there was a possibility that it was not accurate. Fourth, the names of daily medications were not obtained. Finally, we did not provide IgM treatment against nucleocapsid protein after the second dose; however, the number of COVID-19 patients in Fukushima Prefecture were 1,647 between April 19 and June 6, and no patient was identified in Hirata Village between these dates. Thus, we thought that the elevation of antibody titer was caused by vaccinations. Despite these limitations, the present study provided the evidence on the association between individual factors and both antibody titer and neutralizing activity.

## Conclusion

The IgG against S1 protein antibody titer and neutralizing activity after 21 days from the second vaccination were significantly lower among older individuals, those taking immunosuppressive drug and analgesic and antipyretic drug, and those without muscle and joint pain adverse reaction after the second dose. Further research is required to identify the difference of immune response among individuals.

## Supporting information

S1 Fig Anti-SARS-CoV-2 spike protein antibody titers in healthcare workers before and after two doses of the BNT162b2 vaccine. (PPTX)
