# Supplementary material for: Factors associated with anti-severe acute respiratory syndrome coronavirus 2 (SARS-CoV-2) spike protein antibody titer and neutralizing activity among healthcare workers following vaccination with the BNT162b2 vaccine
Source: PLoS One. 2022 Jun 10;17(6):e0269917. doi: 10.1371/journal.pone.0269917 (PMC9187057; doi:10.1371/journal.pone.0269917)
Supplement: S1 Fig — (PPTX) [file pone.0269917.s001.pptx]

## Slide 1
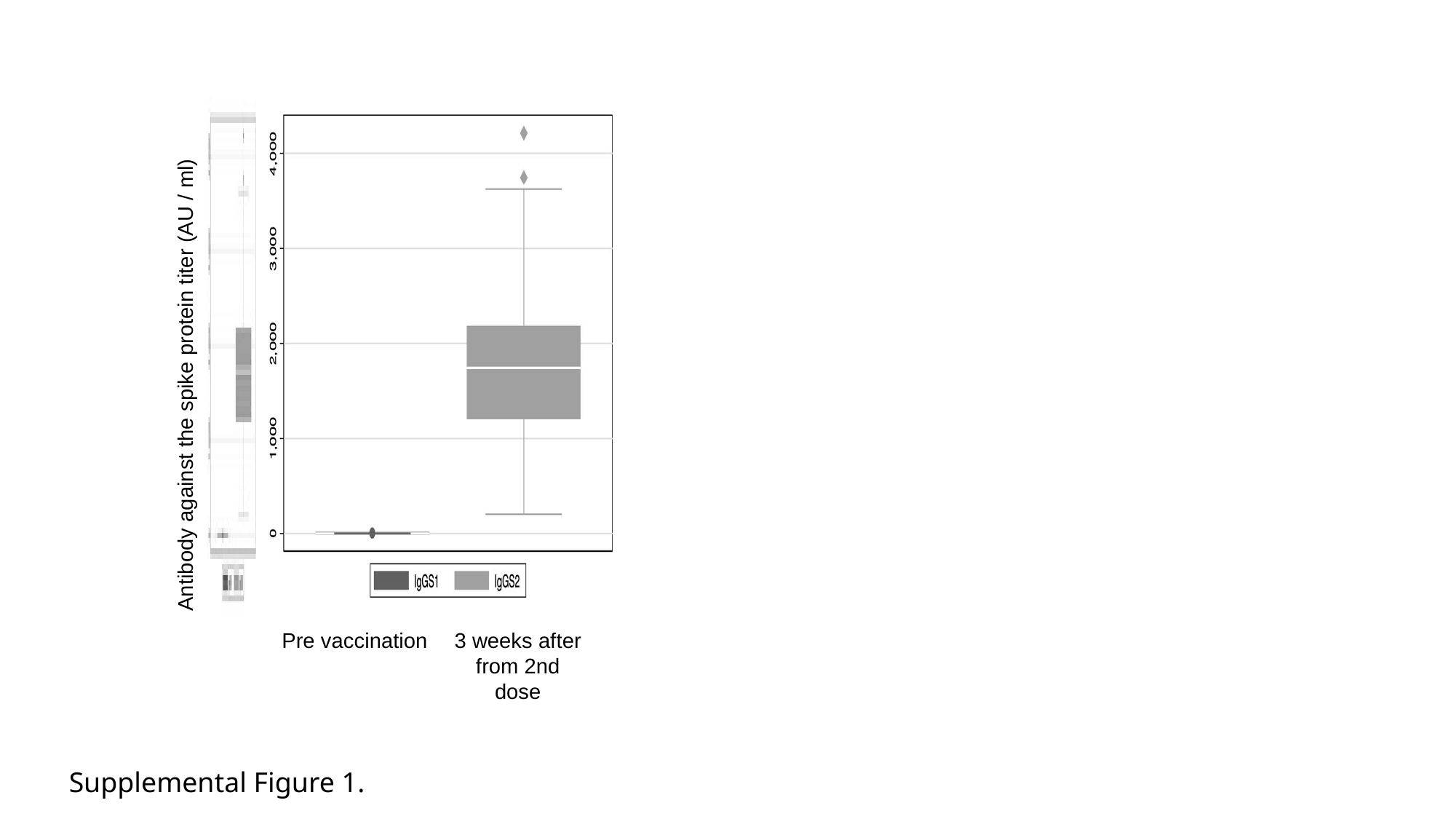

Antibody against the spike protein titer (AU / ml)
Pre vaccination
3 weeks after from 2nd dose
Supplemental Figure 1.
